# Synergistic antimicrobial interaction of plant essential oils and extracts against foodborne pathogens

**DOI:** 10.1002/fsn3.3834

**Published:** 2023-11-22

**Authors:** Manasweeta Angane, Simon Swift, Kang Huang, Janesha Perera, Xiao Chen, Christine A. Butts, Siew Young Quek

**Affiliations:** ^1^ Food Science, School of Chemical Sciences The University of Auckland Auckland New Zealand; ^2^ Faculty of Medical and Health Sciences, School of Medical Sciences The University of Auckland Auckland New Zealand; ^3^ The New Zealand Institute for Plant & Food Research Limited Palmerston North New Zealand; ^4^ Riddet Institute New Zealand Centre of Research Excellence for Food Research Palmerston North New Zealand

**Keywords:** antimicrobial, essential oil, natural preservative, plant extract, synergistic combinations

## Abstract

Essential oils (EOs) and plant extracts have demonstrated inhibitory activity against a wide range of pathogenic bacteria. In this study, the chemical composition of manuka, kanuka, peppermint, thyme, lavender, and feijoa leaf and peel EOs and feijoa peel and leaf extracts were analyzed, and their antimicrobial activity against *Escherichia coli*, *Salmonella enterica* Typhimurium, *Staphylococcus aureus*, *Bacillus cereus*, and *Listeria monocytogenes* were determined. The results showed that the major compounds varied among different EOs and extracts, with menthol in peppermint EO, thymol and carvacrol in thyme EO, linalool in lavender EO, β‐caryophyllene in feijoa EO, and flavones in feijoa extract being the most prevalent. The study found that while EOs/extracts had antimicrobial activity alone, no individual EO/extract was highly effective against all tested species. Therefore, their combinations were tested to identify those that could broaden the spectrum of activity and act synergistically. The checkerboard method was applied to assess the possible synergism between the paired combinations of EOs/extract. The peppermint/thyme, peppermint/lavender, and peppermint/feijoa peel extract combinations exhibited a synergistic effect against *E. coli* and *L. monocytogenes*, with the peppermint/thyme and peppermint/feijoa peel extract combinations being the most effective against all five pathogens. Time‐to‐kill kinetics assays demonstrated that peppermint/thyme and peppermint/feijoa peel extract combinations achieved complete eradication of *E. coli* within 10–30 min and *L. monocytogenes* within 4–6 h. This study provides a promising approach to developing a natural alternative for food preservation using synergistic combinations of EOs/extracts, which could potentially reduce the required dosage and broaden their application in food products as natural preservatives.

## Graphical abstract

Plant essential oils and extracts possess inhibitory activity against foodborne pathogens. The use of synergistic combination reduced the bactericidal concentration, gave broad‐spectrum activity, potentially minimizing the adverse sensory attributes. This study highlights the use of essential oil and extracts as a natural preservative in food.

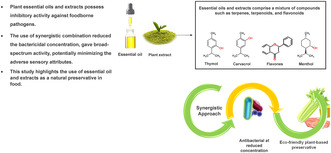

## 1 INTRODUCTION

Foodborne illness is a significant public health issue worldwide, with an estimated 600 million cases reported annually, resulting in 420,000 deaths globally each year (WHO, 2022). In recent years, foodborne diseases have been associated with the emergence of antibacterial‐resistant pathogens, posing an added challenge and paving the way for humankind to enter into an anticipated postantibiotic era (Soulaimani et al., 2021). Resistant foodborne pathogens have been frequently detected in the food chain. They are most likely to enter the food chain during processing, packaging, and distribution, causing food quality degradation and financial loss to the food industry (El amrani et al., 2021). Previous studies have reported the presence of foodborne pathogens, like *Escherichia coli*, *Salmonella* spp., *Listeria monocytogenes*, *Staphylococcus aureus*, and *Bacillus cereus* directly in food or from the food processing environment (Ayari et al., 2020; Karagozlu et al., 2011).

In the food industry, eradication of foodborne pathogens to ensure food safety is achieved mainly by using chemical preservatives, for example, chlorine‐based preservatives, butylated hydroxyanisole (BHA), butylated hydroxytoluene (BHT), and nitrates and nitrites (Santos et al., 2017). However, chemical preservatives have been associated with several side effects, such as nausea, headache, weakness, seizures, mental retardation, and anorexia (Bag & Chattopadhyay, 2015). They are also known for their carcinogenic effects, posing a significant risk to human health (Olmez & Kretzschmar, 2009). Increased awareness of the side effects related to the use of chemical preservatives in food has resulted in an outcry of consumers to develop natural antimicrobial preservatives as an alternative.

Natural substances such as plant essential oils (EOs) and their extracts comprise an extensive reservoir of antimicrobial compounds, which could offer an alternative solution to combat the growth of foodborne pathogens (Soulaimani et al., 2021). Researchers have extensively studied the antimicrobial activity of individual EOs and their plant extracts against various pathogens (Ez zoubi et al., 2020; Qian et al., 2020; Raj et al., 2020; Singh et al., 2018; Yang et al., 2021). However, a closer look at the literature reveals that a single EO or extract requires higher concentrations to accomplish similar effects in situ compared to those established in vitro (Angane et al., 2022; Bassole & Juliani, 2012; Burt, 2004). It is noted that there are disadvantages to this approach, such as negative organoleptic perception (alteration in food's taste and aroma) when higher concentrations of EOs/extracts are present in the food matrix (Khaleque et al., 2016). Another disadvantage is that EOs/extracts have demonstrated strong antimicrobial potential mainly against gram‐positive bacteria, but exert moderate‐to‐weak antimicrobial activity against gram‐negative bacteria (Deng et al., 2020; Soulaimani et al., 2021). A solution to these disadvantages is to combine EOs/extracts using a synergistic approach to inhibit foodborne pathogens (Milagres de Almeida et al., 2023). The advantage of using this approach is that combinations of EOs/extracts will reduce the required total concentration of EOs/extracts, thereby minimizing the undesirable aromas and flavors that they may individually impart to the food (Kim et al., 2021). It will also give a broad spectrum of antimicrobial activity against gram‐positive and gram‐negative bacteria and increase their efficacy in complex food matrices (Cava‐Roda et al., 2021).

Recent research has reported the improved antimicrobial potential of various EO/extract combinations applied at lower doses. For example, Soulaimani et al. (2021) demonstrated synergistic and additive effects of combining lavender, thyme, and rosemary EO against gram‐negative bacteria. Similarly, Chaichi et al. (2021) reported a synergistic and additive activity of the combination of thyme, cinnamon, and clove EO against *E. coli*, *St. aureus*, and *Pseudomonas fluorescens*, and Cava‐Roda et al. (2021) observed synergistic effects of vanillin/clove EOs and vanillin/cinnamon bark EOs against *E. coli* O157:H7 and *L. monocytogenes*. Also, the combination of cinnamon and thyme EOs was identified to have a synergistic anti‐*Listeria* effect in organic tomato juice (Kim et al., 2021). The abovementioned studies demonstrated two‐ to fourfold reductions in the EO concentration when used in combination compared to single treatments.

In light of these studies, it is conceivable that using combinations of EOs/extracts is an effective approach to lowering the concentrations required to have a bacteriostatic or bactericidal effect. Hence, we seek to identify effective combinations of EOs and plant extracts that could exhibit a broad spectrum of activity against foodborne pathogens, utilizing locally sourced materials from New Zealand where possible. We assessed their effectiveness against gram‐negative and gram‐positive organisms as an initial measure of their spectrum. For this purpose, we selected Manuka and Kanuka EOs derived from native New Zealand plants with documented antimicrobial properties (Chen et al., 2016; Fratini et al., 2019; Mathew et al., 2020). To represent less explored plant species in New Zealand with potential antimicrobial applications, we included feijoa peel and leaf EOs and extracts. Additionally, we incorporated peppermint, thyme, and lavender EOs based on the existing literature (Erland & Mahmoud, 2016; Hejna et al., 2021; Lages et al., 2021) and preliminary screening, suggesting their potential activity against either gram‐negative (peppermint EO) or gram‐positive (thyme and lavender EO) bacteria.

This research aims to evaluate the chemical composition and antibacterial activity of commercially available manuka (*Leptospermum scoparium*), kanuka (*Kunzea ericoides*), peppermint (*Mentha piperita*), thyme (*Thymus vulgaris*), lavender (*Lavandula intermedia*) Eos, and laboratory‐derived feijoa (*Acca sellowiana*) EOs (leaf and peel) and their ethanol extract (leaf and peel) against five foodborne pathogens (*E. coli*, *S. enterica* Typhimurium, *St. aureus*, *B. cereus*, and *L. monocytogenes*). Some existing studies in the broader literature have examined the antimicrobial potential of manuka (Mathew et al., 2020), kanuka (Essien et al., 2019), peppermint (Camele et al., 2021; Hejna et al., 2021), thyme (Lages et al., 2021), lavender (Adaszyńska‐Skwirzyńska et al., 2021), feijoa EO and its extracts (Santos et al., 2021; Smeriglio et al., 2019) individually, but the effect of combining these EOs as well as feijoa extracts has not been established. Feijoa is a subtropical fruit widely cultivated in many parts of the world, including Australia and New Zealand (Bell et al., 2018). Despite studies on its antimicrobial (Phan et al., 2019), antioxidant (Peng et al., 2019b), anticancer (Bontempo et al., 2007), and anti‐inflammatory activities (Peng et al., 2018), there has been little discussion on its potential synergistic applications as a natural preservative.

Our primary goal was to identify a synergistic or additive combinations that are active against gram‐negative and gram‐positive bacteria. In this context, we evaluated the susceptibility of *E. coli* and *L. monocytogenes* to paired combinations of EOs/extracts to detect synergistic, additive, indifferent/no interaction, or antagonistic effects in vitro. Synergistic combinations were also investigated for bactericidal effects against *St. aureus*, *B. cereus*, and *S. enterica* Typhimurium to ensure broad‐spectrum activity using the proposed combinations, and time‐to‐kill kinetics was performed to validate the efficacy of EO/extract combinations. The outcomes of this study are expected to benefit the food industry by bringing a new class of natural plant‐based preservatives with a broad spectrum of antimicrobial activities.

## 2 MATERIALS AND METHODS

### 2.1 Sample collection and storage

Feijoa fruit and leaf samples were handpicked from regional orchards located in the Ngatea region of the North Island of New Zealand. The fruit was collected during the peak season from February to March 2021, and leaves were collected during the pruning season from May to June 2021. The feijoa fruits and leaves were transported to the laboratory, where they were thoroughly washed under running tap water to remove excess dust, debris, and mud from the surface. The feijoa fruit was peeled manually, and the leaves were air‐dried, then packed in ziplock bags and stored at −20°C until analysis. Manuka and kanuka EOs were purchased from New Zealand Manuka Bioactives Ltd., Opotiki, New Zealand and Pure Nature, Auckland, New Zealand, respectively. Peppermint, thyme, and lavender EOs were provided by Pure Ingredients, Glendene, New Zealand.

### 2.2 Extraction of feijoa peel and leaf EO

The feijoa peel was cut into small pieces, and air‐dried feijoa leaves were ground to a fine powder using a Waring blender (Vitamix, Cleveland, OH). The peels and the ground leaves were subjected to hydrodistillation for 8 h, using Clevenger‐type apparatus, as described by Peng et al.'s (2019a) study. The EO was then transferred to an airtight, amber‐colored glass vial, flushed with nitrogen gas, and stored at −80°C until analysis.

### 2.3 Preparation of feijoa peel and leaf ethanol extract

Ethanol extraction was conducted according to Peng et al. (2018). The peel and leaf samples were transferred to −80°C storage for at least 8 h and then freeze‐dried for 72 h (Labconco freeze dryer, USA). The freeze‐dried peel and leaf samples were stored at −80°C in an airtight container before extraction. For the fruit peels, 20 g of the freeze‐dried powder was mixed with 1 L of 50% (v/v) ethanol and then extracted for 45 min using a magnetic hot plate stirrer set at 60°C and 600 rpm. For the feijoa leaves, 20 g of freeze‐dried powder was mixed with 1 L of 30% (v/v) ethanol and then extracted for 10 min using a magnetic hot plate stirrer set at 50°C and 600 rpm.

### 2.4 Chemical characterization

Gas chromatography–mass spectrometry (GC–MS) was used to determine the chemical composition of EO, as described by Peng et al. (2019a) with some modifications. Briefly, 1 μL of feijoa peel and leaf EO was dissolved in 1 mL hexane and 10 μL of internal standard (2‐methyl‐3‐heptanone, 312 μg/mL) was added. The GC–MS (Agilent 7890A) used was coupled to a DB‐5MS column (30 m × 250 μm × 0.25 μm). Helium was used as the carrier gas at a 1.5 mL/min flow rate. The sample was injected using splitless mode at a temperature of 250°C. The oven temperature was maintained at 35°C for 0 min, followed by an increase at 8°C/min to 105°C, then 3°C/min to 150°C, and the final ramp was set to increase at a rate of 25°C/min to 280°C. The ion source temperature was set at 200°C, with electrons generating at 70 eV with a scanning range from 41 to 500 *m*/*z*. The volatile compounds in EO were identified by using the NIST Mass Spectral Library. Liquid chromatography–mass spectrometry (LC–MS) was used to determine the chemical composition of feijoa extracts, as described by Peng et al. (2019b) without any modifications.

### 2.5 Bacterial cultures

The EOs and extracts were tested against 2 g‐negative bacteria, *E. coli* ATCC 25922 and *S. enterica* Typhimurium ATCC 10702, and 3 g‐positive bacteria, *St. aureus* ATCC 6538, *B. cereus* ATCC 11778, and *L. monocytogenes* CDC H2446. These cultures were obtained from the New Zealand Clinical Collection Culture Collection held by the Institute of Environmental Science and Research (ESR).

### 2.6 Determination of minimum inhibitory concentration (MIC) and minimum bactericidal concentration (MBC)

The MBC of commercially available manuka, kanuka, peppermint, thyme, and lavender EOs and laboratory‐derived feijoa EOs (leaf and peel) and ethanol extract (leaf and peel) was determined by using the double dilution method (Beikzadeh et al., 2020). A stock solution of each EO/extract was prepared at a maximum concentration of 400 mg/mL (40%) by dissolving 400 mg of EO/extract in 600 μL of absolute ethanol (Liu et al., 2020). The stock solutions were further diluted at a ratio of 1:1 with Mueller–Hinton broth (MHB; Difco™, Becton, Dickinson & Co.) + 1% Tween 80 (Sigma‐Aldrich). The final concentration of each working stock solution was 200 mg/mL of EO/extract (20%) and 30% of ethanol. In a 96‐well plate, 100 μL of homogenized EO/extract solution was added to the first row, and a twofold serial dilution was carried out with MHB. A 50‐μL of bacterial suspension prepared in MHB adjusted to approximately 1 × 10^6^ CFU/mL was then dispensed into each well. The last row was treated as a positive control. The plates were incubated at 37°C for 24 h in a shaking incubator operating at 200 rotations per minute (rpm) in a sealed box humidified with moistened tissue paper. MIC was determined as the lowest concentration at which no visible growth occurred. MBC was determined by plating 10 μL from each well on the Mueller–Hinton agar (MHA; Difco™) plate, and the plate was incubated at 37°C for 24 h. MBC was determined as the lowest concentration at which no bacterial colonies were observed, indicating a >1000‐fold reduction in the number of viable cells remaining after the test period (Appendix S1; European Committee for Antimicrobial Susceptibility Testing [EUCAST], 2000). The EOs and extracts tested contributed to the turbidity of the medium; hence, MIC was not recorded.

### 2.7 Synergistic assay

To determine antimicrobial synergy between EO and extracts, the stock solutions were prepared at the maximum concentration of 4× MBC in ethanol. Two compounds were tested per assay and labeled Compound A and Compound B. The stock solution containing Compound A was added to the first row of Plate 1, and the stock solution containing Compound B was added to all wells in the last column of Plate 2. Twofold dilutions were carried out in MHB, and the last row and column were maintained as a positive control. Furthermore, 25 μL was withdrawn from the corresponding wells of both plates and mixed gently in the matched well of the third plate (Appendix S2). The bacterial suspensions adjusted to a concentration of 10^6^ CFU/mL was added to all wells of Plate 3. Plates were incubated at 37°C for 24 h. The drop plate was performed on the MHA plate to assess bactericidal concentrations and combinations. The fractional bactericidal concentration (FBC) index was calculated using the following formula:

$${\mathrm{FBC}}_{A}=A/{\mathrm{MBC}}_{A}$$

$${\mathrm{FBC}}_{B}=B/{\mathrm{MBC}}_{B}$$

(1) $${\mathrm{FBC}}_{\text{Index}}={\mathrm{FBC}}_{A}+{\mathrm{FBC}}_{B}$$

where A and B are the MBC of each compound in combination, and MBC_A_ and MBC_B_ are the MBC of individual compounds. Interactions between EO/extracts were interpreted as follows: synergistic (<0.5), additive (>0.5–1), indifferent/no interaction (>1–4), and antagonistic (>4) (Bag & Chattopadhyay, 2015). An isobologram was then plotted (Appendix S2) to depict the hypothetical scenario of synergistic, additive, and antagonistic interactions (EUCAST, 2000).

### 2.8 Time‐to‐kill kinetic

A time‐to‐kill assay was conducted to assess the effectiveness of synergistic combinations of EOs and extracts with the lowest FBC index as per the method described by Lim et al. (2023). Bovine serum albumin (BSA) was added to the samples to mimic the protein burden in the food matrix. Samples were prepared at FBC, ½ FBC, and ¼ FBC concentrations in MHB, and a bacterial suspension was added to achieve a concentration of 1 × 10^7^ CFU/mL. A positive control consisting of a bacterial suspension in MHB without EO/extract was also included. The control and samples were incubated at 37°C with shaking at 200 rpm, and aliquots were taken at 10 min, 30 min, and 1, 2, 4, 6, and 8 h. Serial 10‐fold dilutions were performed in the letheen broth to deactivate the EO/extract, and then appropriate dilutions were plated on MHA. After incubating the plates at 37°C for 24 h, the number of surviving bacteria was enumerated.

### 2.9 Statistical analysis

All measurements were performed in triplicate, and median values were plotted for the antimicrobial assays. Analysis of variance (ANOVA) via Sigmaplot (version 13.5, Systat Software Inc.) was used to analyze the experimental data statistically. Tukey's honestly significant difference test was used to compare the means obtained by time‐to‐kill kinetics at a preset significance level of *p <* .05.

## 3 RESULTS AND DISCUSSION

### 3.1 Chemical characterization

The chemical composition analysis of the tested EOs are presented in Table 1. The primary constituents of manuka EO were β‐triketone, which includes leptospermone (222.25 μg/μL), isoleptospermone (63.64 μg/μL), *cis*‐calamenene (182.04 μg/μL), and flavesone (56.56 μg/μL). The manuka EO also contains sesquiterpenes and sesquiterpene hydrocarbons in minor quantities. Douglas et al. (2004), Mathew et al. (2020), and Melching et al. (1997) have previously reported that β‐triketone formed the major constituent of manuka EO, and we now confirm this finding. The kanuka EO, however, exhibited a higher concentration of monoterpene compounds, with α‐pinene (1212.13 μg/μL) being the most abundant, followed by *o*‐cymene (127.91 μg/μL), γ‐terpinene (43.19 μg/μL), and β‐pinene (10.18 μg/μL). Unlike the manuka EO, β‐triketone and sesquiterpenes did not constitute a significant proportion of the kanuka EO. This finding corroborates the results reported by Essien et al. (2019), who highlighted the main differences between manuka and kanuka EO as the concentrations of β‐triketone and α‐pinene, respectively.

**TABLE 1:** Quantitative analysis of chemical composition of manuka, kanuka, feijoa peel, feijoa leaf, peppermint, thyme, and lavender EO.

CRI	RI	Compounds	Relative concentration (μg/μL)						
			Manuka	Kanuka	Feijoa peel	Feijoa leaf	Peppermint	Thyme	Lavender
875	865	3‐Methylbutyl acetate		4.42 ± 0.13					
931	933	α‐Pinene	12.69 ± 1.22	1212.13 ± 73			17.04 ± 1.84	42.77 ± 5.92	
947	947	Camphene		2.51 ± 0.15				15.19 ± 2.35	2.81 ± 0.33
971	968	β‐Thujene				1.86 ± 0.15			
975	978	Sabinene				0.95 ± 0.04	5.23 ± 0.63	3.55 ± 0.71	1.06 ± 0.14
975	974	β‐Pinene	1.32 ± 0.17	10.18 ± 0.44			23.93 ± 1.87		
979	979	1‐Octen‐3‐ol							3.22 ± 0.53
983	988	3‐Octanone			9.45 ± 0.28	25.64 ± 1.35			4.83 ± 0.58
988	985	β‐Myrcene	5.11 ± 0.44			2.45 ± 0.01		12.28 ± 2.32	48.98 ± 3.15
994	990	Butyl butanoate							1.95 ± 0.18
996	995	3‐Octanol			2.99 ± 0.11	2.99 ± 0.37	5.67 ± 0.53		
1004	1005	α‐Phellandrene				5.8 ± 0.39			
1006	1009	3‐Carene							8.32 ± 0.77
1011	1010	Hexyl acetate							7.03 ± 0.61
1014	1004	(+)‐4‐Carene				1.31 ± 0.14			1.73 ± 0.18
1015	1057	Isoterpinolene		1.45 ± 0.16			0.5 ± 0.14		
1023	1018	*o*‐Cymene	1.66 ± 0.12	127.91 ± 6.37		6.39 ± 0.66	8.1 ± 0.74	500.97 ± 71.71	2.86 ± 0.36
1028	1028	D‐Limonene					67.63 ± 8.91	13.81 ± 2.42	21.27 ± 0.92
1029	1031	β‐Phellandrene				113.35 ± 6.98			
1031	1038	Eucalyptol		134.83 ± 7.05			106.41 ± 7.61		
1035	1041	trans‐β‐Ocimene			13.3 ± 0.05	3.81 ± 0.3			80.01 ± 6.56
1046	1043	β‐Ocimene			2.14 ± 0.09	0.85 ± 0.01			48.59 ± 3.87
1057	1055	γ‐Terpinene	1.37 ± 0.08	43.19 ± 2.28		1.4 ± 0.14	0.86 ± 0.09		2.11 ± 0.24
1069	1072	*Trans*‐Linalool oxide (furanoid)							1.63 ± 0.2
1084	1086	Terpinolene						1.56 ± 0.34	4.2 ± 0.46
1098	1091	Linalool		37.88 ± 2.86	70.32 ± 1.56	92.44 ± 9.69		117.68 ± 20.72	505.84 ± 33.38
1104	1105	3‐Methylbutyl isovalerate	2.66 ± 0.1						
1105	1110	Octenyl acetate							20.9 ± 1.27
1104	1103	Isoamyl isovalerate		2.14 ± 0.14					
1112	1115	3‐Methylbut‐3‐enyl isovalerate	3.01 ± 0.23						
1114	1115	Fenchol						5.74 ± 1.16	
1121	1126	α‐Campholenal		4.87 ± 0.49					
1122	1120	Alloocimene							10.51 ± 0.76
1123	1131	*Neo*‐Allo‐Ocimene			1.54 ± 0.07				
1129	1134	1‐Terpineol						9.05 ± 1.6	
1134	1139	(−)‐Trans‐Pinocarveol		6.97 ± 0.36					
1139	1141	(+)‐2‐Bornanone						5.91 ± 0.9	2.98 ± 0.23
1141	1149	Isopulegol					2.18 ± 0.22		
1142	1153	*Cis*‐β‐Terpineol						10.07 ± 1.78	
1148	1166	(+)‐Isomenthone					543.72 ± 42.28		
1156	1147	Menthofuran					22.17 ± 2.38		
1156	1170	Lavanduol							18.35 ± 1.41
1156	1156	Isoborneol						10.22 ± 1.73	
1157	1159	(−)‐Isomenthone					84.88 ± 8.95		
1160	1155	*Cis*‐β‐Terpineol						3.77 ± 0.6	
1163	1194	Neoisomenthol					77.17 ± 7.61		
1163	1156	*p*‐Menthan‐1‐ol					70.51 ± 4.35		
1165	1165	Endo‐Borneol						23.91 ± 3.91	13.17 ± 1.03
1172	1171	dl‐Menthol					951.12 ± 74.99		
1174	1179	Terpinen‐4‐ol							33.1 ± 2.87
1175	1175	(−)‐4‐Terpineol		8.54 ± 0.54				4.49 ± 0.81	
1180	1183	Crypton							2.01 ± 0.17
1184	1194	Neoisomenthol					5.77 ± 0.57		
1187	1188	Hexyl butanoate							6.71 ± 0.59
1188	1171	Levomenthol					3.39 ± 0.53		
1191	1190	α‐Terpineol		29.42 ± 1.98		1.94 ± 0.1	7.44 ± 0.79	236.1 ± 37.16	13.36 ± 1.2
1197	1178	γ‐Terpineol						33.08 ± 5.41	
1213	1210	(−)‐*Cis*‐Carveol		2 ± 0.46					
1228	1219	Pulegone					26.11 ± 2.68		
1241	1248	Linalyl acetate							390.53 ± 25.45
1243	1254	Piperitone					6.51 ± 0.68		
1244	1224	Phenethyl acetate		1.57 ± 0.11					
1277	1273	Lavandulyl acetate							120.34 ± 9.15
1277	1328	Nerol acetate							106.05 ± 8.48
1283	1290	Thymol						657.21 ± 102.88	
1283	1294	Menthyl acetate					138.78 ± 18.7		
1288	1297	2‐Undecanone			9.14 ± 0.19	8.52 ± 0.84			
1292	1299	O‐Thymol						148.92 ± 23.41	
1322	1344	Elemene isomer			21.1 ± 0.74	30.48 ± 3.67			
1335	1339	α‐Cubebene	54.38 ± 4	3.97 ± 0.36	44.89 ± 1.42	42.49 ± 4.15			
1348	1347	Nerol acetate							12.61 ± 0.87
1355	1361	Ylangene	2.98 ± 0.67						
1362	1377	Copaene	94.04 ± 6.78	13.78 ± 1.01	8.12 ± 0.51	8.84 ± 0.63			
1369	1363	Geranyl acetate							25.78 ± 1.2
1370	1384	(−)β‐Bourbonene			12.61 ± 0.25	13.88 ± 1.17			
1378	1385	(−)‐*Cis*‐β‐Elemene	10.6 ± 0.74		82.18 ± 0.69	52.41 ± 5.05			
1396	1423	(−)‐Aristolene	13.56 ± 1.07						
1397	1408	(−)α‐Gurjunene		7.94 ± 0.55	21.66 ± 0.97	20.49 ± 1.69			
1406	1418	Caryophyllene	35.19 ± 2.36		208.98 ± 4.23	258.43 ± 24.5	34.81 ± 3.33	20.72 ± 3.28	31.5 ± 2.9
1408	1420	Santalene							5.32 ± 0.78
1414	1428	β‐Copaene			7.08 ± 1.6	6.83 ± 0.29			
1420	1462	Aromandendrene	24.4 ± 2.24			12.58 ± 1.62			
1429	1458	Cadina‐3,5‐diene	74.25 ± 5.53						
1434	1455	Humulene			56.17 ± 1.54	114.65 ± 13.29			
1437	1462	Alloaromadendrene	9.3 ± 0.79		13.3 ± 0.54				
1437	1455	(E)‐2‐Epi‐β‐caryophyllene		12.52 ± 0.76		28.19 ± 2.9			
1441	1445	(E)‐β‐Famesene							15.05 ± 1.12
1451	1458	Cadina‐3,5‐diene	59.96 ± 3.58						
1455	1463	γ‐Muurolene	17.2 ± 1.51			8.59 ± 1.6			
1458	1473	Germacrene D			104.46 ± 2.79	8.59 ± 1.6			
1465	1464	β‐Selinene	68.15 ± 6.43		10.51 ± 1.17				
1469	1476	Ledene		32.23 ± 2.04	32.11 ± 0.37	44.59 ± 4.64			
1473	1463	α‐Selinene	60.12 ± 6.21						
1474	1482	γ‐Elemene			85.11 ± 6.92				
1474	1488	Bicyclogermacrene				125.02 ± 12.86			
1481	1480	α‐Amorphene				14.62 ± 0.11			
1496	1513	(+)‐γ‐Cadinene				12.45 ± 1.54			
1506	1519	Cadina‐1(10),4‐diene			51.13 ± 1.74	54.88 ± 5.68			
1507	1511	*Cis*‐Calamenene	182.04 ± 8.37	46.43 ± 2.19					
1508	1516	*Trans*‐Calamenene			48.93 ± 0.24	40.61 ± 5.66			
1522	1525	1,4‐Cadinadiene	91.5 ± 6.59			14.17 ± 0.92			
1531	1538	α‐Calacorene	8.72 ± 0.66						
1535	1546	Flavesone	56.56 ± 5.81						
1565	1572	(−)‐Spathulenol	6.7 ± 0.3	12.62 ± 0.93	54.38 ± 1.81	65.02 ± 7.07			
1568	1578	Caryophyllene oxide			17.41 ± 0.42	17.88 ± 2.35			
1572	1580	(−)‐Globulol			20.55 ± 0.31	28.39 ± 4.39			
1578	1587	Himbaccol		26.76 ± 1.74	8.52 ± 0.44				
1585	1586	Ledol		13.08 ± 1.02		11.3 ± 1.67			
1598		Isoleptospermone	63.64 ± 6.31						
1606	1620	Leptospermone	222.25 ± 17.25						
1610	1619	(−)‐Spathulenol			15.66 ± 0.88	16.89 ± 1.8			
1619	1623	Epicubenol	16.54 ± 1.43						
1633	1639	α‐Cadinol			23.12 ± 0.58	20.61 ± 1.42			

*Note*: All experiments were performed in triplicate and results were recorded as mean ± standard deviation.

Peppermint EO is composed primarily of monoterpene and monoterpenoid compounds, which comprise up to 90% of its total constituents. The two most abundant volatile components in peppermint EO are menthol, present at a concentration of 951 μg/μL, followed by isomenthone at 628.6 μg/μL. Other compounds present in smaller amounts include menthyl acetate (138.78 μg/μL), eucalyptol (106.41 μg/μL), neoisomenthol (77.17 μg/μL), *p*‐menthan‐1‐ol (70.51 μg/μL), and d‐limonene (67.63 μg/μL). The primary constituents of peppermint EO, menthol, and isomenthone have been previously reported by Bassole et al. (2010) and Moetamedipoor et al. (2021) and are consistent with our findings.

Similarly, thyme EO and lavender EO were also found to be rich in monoterpene compounds. Thyme EO contained thymol (806.13 μg/μL) and carvacrol (521.21 μg/μL) as major components, while lavender EO contained linalool (505.84 μg/μL), linalyl acetate (390.53 μg/μL), lavandulyl acetate (390.53 μg/μL), and nerol acetate (118.66 μg/μL). These results agree with those reported by Ruzauskas et al. (2020), where thymol and carvacrol were the major compounds that were detected in thyme EO.

Our study agreed with observations of Peng et al. (2019a) whereby feijoa peel EO is rich in terpene compounds, including β‐caryophyllene (208.98 μg/μL), germacrene D (104.46 μg/μL), γ‐elemene (85.11 μg/μL), *cis*‐β‐elemene (82.18 μg/μL), humulene (56.17 μg/μL), *trans*‐calamenene (48.93 μg/μL), α‐cubebene (44.89 μg/μL), and ledene (32.11 μg/μL). We also found other terpene alcohols, including linalool (70.32 μg/μL), spathulenol (54.38 μg/μL), α‐cadinol (23.12 μg/μL), and globulol (20.55 μg/μL). Our study findings are also consistent with those of Fernandez et al. (2004) and Elfarnini et al. (2018), except for some minor differences in some of the chemical compounds present, which is likely attributed to the differences in the cultivar and extraction methods used in the different studies. Feijoa leaf EO shared similarities with feijoa peel EO, with β‐caryophyllene being the main terpene compound identified. This has been previously reported by Kong (2019) who observed that the chemical composition of the feijoa leaf EO was comparable to that of the peel EO. Nevertheless, flavones were the primary phenolic compound detected in both feijoa leaf and peel extracts, followed by procyanidin B1, epicatechin, quercitrin 3‐d‐galactoside, procyanidin B2, epicatechin gallate (ECG), and ellagic acid (Table 2).

**TABLE 2:** Concentration of phenolic compounds in feijoa peel and leaf.

Number	Product name	Feijoa peel (μg/mL)	Feijoa leaf (μg/mL)
1	Gallic acid	5.73 ± 0.39	1.28 ± 0.23
2	Catechin hydrate	757.86 ± 0.72	389.72 ± 0.43
3	2,4‐Dihydroxybenzoic acid	1.75 ± 0.49	0.91 ± 0.29
4	3‐Hydrobenzoic acid	1.24 ± 0.14	1.28 ± 0.08
5	Ellagic acid	193.01 ± 0.13	83.37 ± 0.08
6	Epigallocatechin (EGC)	13.03 ± 0.09	10.35 ± 0.05
7	Epigallocatechin gallate	17.81 ± 0.14	1.11 ± 0.08
8	Epicatechin gallate (ECG)	172.15 ± 0.08	134.96 ± 0.04
9	Quercitin‐3‐glucoside	4.86 ± 0.10	1.90 ± 0.06
10	Quercetin	27.93 ± 13.71	8.53 ± 8.23
11	Quercitrin 3‐d‐galactoside	406.32 ± 1.82	210 ± 1.09
12	Isoquercetin (quercetin 3‐glucoside)	10.95 ± 2.34	4.97 ± 1.40
13	Myricetin	6.97 ± 4.48	3.16 ± 2.68
14	Flavone	9228.26 ± 3.33	2442.19 ± 1.99
15	Procyanidin B1	1275.47 ± 3.79	204.44 ± 2.27
16	2,5‐Dihydrobenzoic acid	3.18 ± 0.59	2.70 ± 1.77
17	Procyanidin B2	360.13 ± 2.16	189.19 ± 1.29
18	Protocatechuic acid	16.53 ± 2.02	10.79 ± 1.21
19	Rutin	102.60 ± 2.72	33.94 ± 1.63
20	4‐Hydroxybenzoic acid	9.38 ± 2.21	3.12 ± 2.21

*Note*: All experiments were performed in triplicate and results were recorded as mean ± standard deviation. Refer Appendix S3 for quantification method of phenolic compounds.

### 3.2 Antimicrobial activity of EOs and extracts

This assay aimed to assess the antimicrobial properties of manuka, kanuka, peppermint, thyme, lavender, feijoa EOs, and feijoa extracts against five foodborne pathogens. We observed that the EOs demonstrated varying degrees of antimicrobial activity against the species tested, ranging from moderate (5% or 5 mg/mL) to strong (<5% or 5 mg/mL), with a few cases exhibiting only very weak activity (>5% or 5 mg/mL). Lower MBC values indicate higher efficacy of EO/extract against foodborne pathogens. The tested EOs exhibited high antimicrobial activity against *St. aureus*, *B. cereus*, while showing moderate‐to‐low inhibitory activity against *L. monocytogenes*, *E. coli*, and *S. enterica* Typhimurium (Table 3).

**TABLE 3:** Antimicrobial activity of tested EOs and extracts against *Escherichia coli*, *Salmonella enterica* Typhimurium, *Staphylococcus aureus*, *Bacillus cereus*, and *Listeria monocytogenes*. The MBC values are presented in mg/mL.

Bacterial pathogens	Feijoa peel		Feijoa leaf		Manuka	Kanuka	Peppermint	Thyme	Lavender
	EO	EX	EO	EX					
*E. coli* ATCC 25922	50	25	50	50	100	100	6.25	12.5	12.5
*S. enterica* ATCC 10702	100	25	100	25	100	100	50	50	50
*St. aureus* ATCC 6538	6.25	25	3.125	1.5	1.5	25	25	25	50
*B. cereus* ATCC 11778	3.125	6.25	1.5	6.25	0.7	6.25	12.5	25	12.5
*L. monocytogenes* CDC H2446	6.25	12.5	3.125	25	0.7	25	25	25	25

*Note*: All experiments were performed in triplicate and median values were recorded. The EOs and extracts contributed to the turbidity of medium, hence MIC was not recorded.

Abbreviations: EO, Essential oil; EX: Extract.

Manuka and kanuka EOs were effective against gram‐positive bacteria. However, a higher concentration of 10% (10 mg/mL) was required for both manuka and kanuka EOs to demonstrate antibacterial effect against gram‐negative bacteria. Similarly, feijoa leaf and peel EOs showed inhibitory effects against *E. coli* and *S. enterica* Typhimurium only at concentrations of 5% (5 mg/mL) and 10% (10 mg/mL), respectively. Based on the MBC values, *B. cereus* was the most sensitive to the manuka and kanuka EOs. This is consistent with other studies which also found that *St. aureus*, *Bacillus* spp., and *L. monocytogenes* were more susceptible to *Cudrania tricuspidata* fruit EO (Bajpai et al., 2013) and grapefruit EO (Deng et al., 2020) compared to gram‐negative bacteria such as *E. coli*, *Salmonella* Typhimurium and *Pseudomonas aeruginosa*. The difference in the susceptibility could be attributed to the gram‐negative bacteria having a more rigid and intricate outer membrane with an abundance of lipopolysaccharides compared to gram‐positive bacteria, which might restrict the dispersion of hydrophobic molecules through the cell wall of the bacteria (Angane et al., 2022).

In contrast, in gram‐positive bacteria, the complex outer membrane is absent. Instead, they have a thick peptidoglycan layer comprising the lipophilic tail of the lipoteichoic acid, which might ease the entry of hydrophobic molecules such as EOs into the cell wall (Chouhan et al., 2017). Some authors have speculated that high levels of β‐ triketones in the manuka EO could be responsible for its inhibitory activity. Although Chen et al. (2016) found that kanuka and manuka EOs were equally effective at inhibiting *E. coli* and *St. aureus*, this study found that kanuka EO exhibited poor inhibitory effects on all the tested pathogens. The reason for this rather contradictory result could be the larger molecular size of EO, which may limit the compound passing through the bacterial cell membrane (Van de Vel et al., 2019). Another possible reason may be due to the effect of different varieties and sources of kanuka trees (Mathew et al., 2020).

Furthermore, the findings in this study agree with those of previous studies examining the effect of feijoa peel EO on *St. aureus* and *E. coli* (Smeriglio et al., 2019). They reported a MIC of 2.67 mg/mL, MBC of 5.35 mg/mL against *St. aureus*, and no activity against *E. coli*. In the current study, the MBC value against *St. aureus* for feijoa peel EO was reported as 0.312% (3.12 mg/mL) and 0.625% (6.25 mg/mL) and for leaf EO as 0.15% (1.5 mg/mL) and 0.312% (3.12 mg/mL), respectively, which are in agreement with those of Smeriglio et al. (2019). The presence of β‐caryophyllene in feijoa EO may contribute to its inhibitory activity (Basile et al., 2010).

Peppermint, thyme, lavender EOs, and the feijoa extracts, however, followed a different trend, and their effect was random against gram‐positive and gram‐negative bacteria. Peppermint EO displayed a potent antibacterial effect against gram‐negative *E. coli* at a concentration of 0.6% (6.25 mg/mL), exhibiting a strong activity. The primary contributors to the antibacterial activity of these EOs/extracts are expected to be menthol in peppermint EO (Bassole et al., 2010), thymol and carvacrol in thyme EO (Chaichi et al., 2021), linalool in lavender EO (Garzoli et al., 2020), and flavone in feijoa peel extract (Peng et al., 2019b). These results align with the study by Trombetta et al. (2005), where menthol, the major component of peppermint EO, was found to be more effective against *E. coli* than other terpene compounds tested. The effectiveness of thyme EO can be attributed to the hydrophobic nature of the thymol and carvacrol molecules (Chaichi et al., 2021), which can disrupt the outer membrane of gram‐negative bacteria. This disruption results in the release of lipopolysaccharides and an increase in the permeability of the cytoplasmic membrane (Bassole et al., 2010). Furthermore, Motohashi et al. (2000) and Phan et al. (2019) reported the potent antibacterial activity of feijoa peel extract against *E. coli*, *P. aeruginosa*, *St. epidermidis*, and *St. aureus*. However, in their study, the agar disk diffusion method was used to evaluate the efficacy of the peel extracts. The agar disk diffusion method is a quick typing tool to determine the sensitivity of bacteria but cannot differentiate between bacteriostatic and bactericidal effects (Angane et al., 2022). This is the first publication to report the antibacterial activity of EOs of feijoa and ethanol extract of feijoa using the MBC assays.

### 3.3 Synergistic effects of EO and extracts on selected bacteria

The use of synergism can be an effective strategy for reducing the bactericidal concentration required for reducing foodborne pathogens. The primary objective of this study was to develop a broad‐spectrum approach for targeting these pathogens while also improving the antibacterial interactions between EOs and extracts and minimizing any associated adverse sensory attributes. Various binary combinations were evaluated using the checkerboard method to achieve these objectives, and the fractional bactericidal concentration (FBC) index was determined. Given that meat products are commonly contaminated with gram‐negative pathogens, manuka and kanuka EO were excluded from the synergistic study, as they require higher concentrations (10% or 10 mg/mL) to exhibit antibacterial activity against gram‐negative bacteria. Peppermint EO was the most potent against gram‐negative bacteria from our findings, so it was chosen for testing in combination with thyme, lavender, feijoa peel and feijoa leaf EO, feijoa peel extract, and feijoa leaf extract. Furthermore, we focused our synergistic study on two model organisms: *E. coli* to represent gram‐negative bacteria and *L. monocytogenes* to represent gram‐positive bacteria.

#### 3.3.1 Synergistic effect of selected EOs and extracts

**FIGURE 1:**
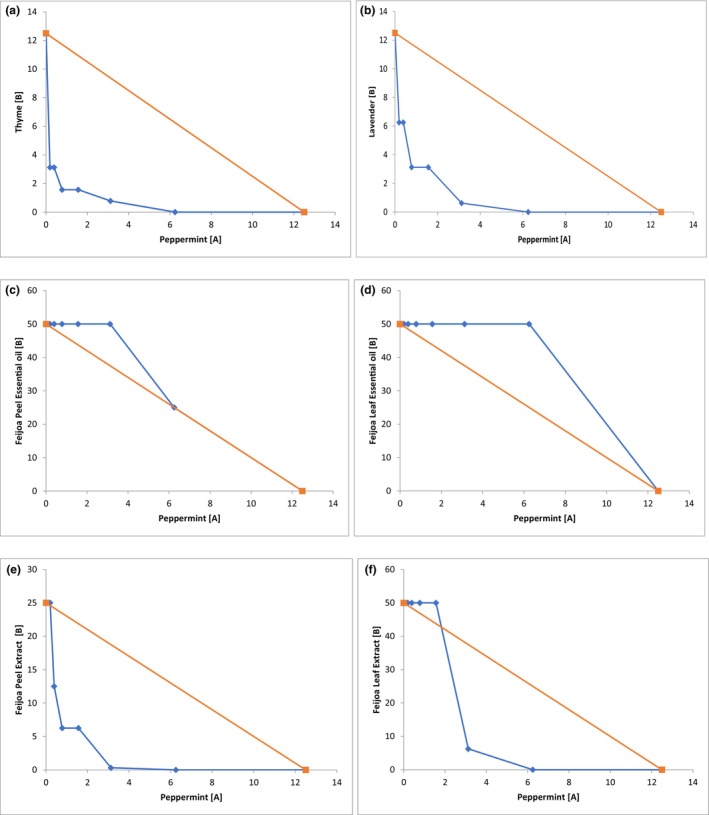
Isobolograms of selected combinations of EO and extracts against *E. coli*. The FBC values on the X and Y axes are represented in mg/mL. FBC values of compound A and compound B (blue) are shown with the theoretical line (orange) for an additive interaction. Compound A is Peppermint EO, and Compound B is Thyme EO (a); Lavender EO (b); Feijoa Peel EO (c); Feijoa Leaf EO (d); Feijoa Peel Extract (e); and Feijoa Leaf Extract (f). (Key: 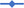: FBC values of compound A and compound B; 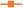: Additive line).

**FIGURE 2:**
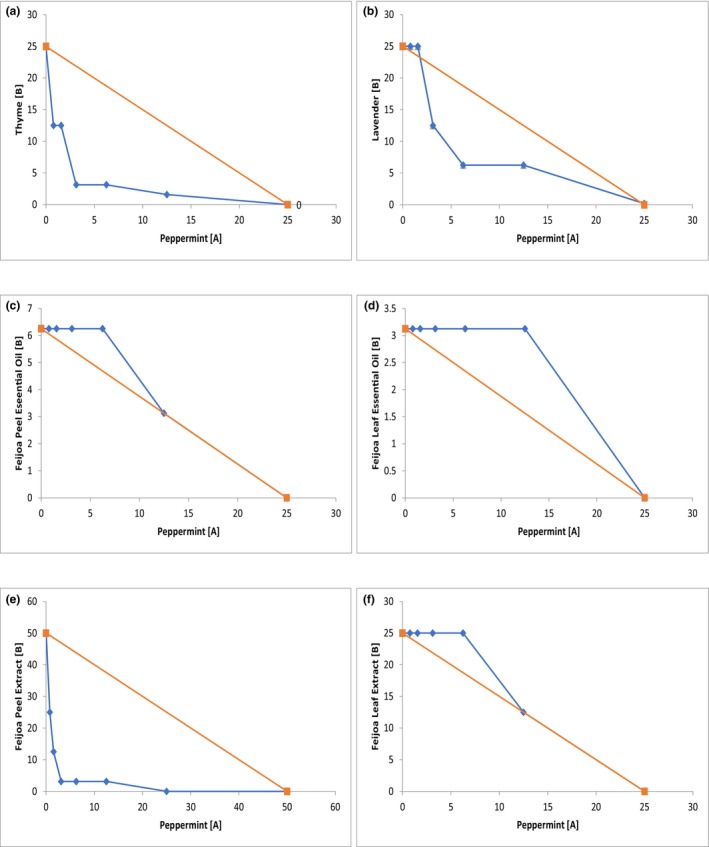
Isobolograms of selected combinations of EO and extracts against *L. monocytogenes*. The FBC values on the X and Y axes are represented in mg/mL. FBC values of compound A and compound B (blue) are shown with the theoretical line (orange) for an additive interaction. Compound A is Peppermint EO, and Compound B is Thyme EO (a); Lavender EO (b); Feijoa Peel EO (c); Feijoa Leaf EO (d); Feijoa Peel Extract (e); and Feijoa Leaf Extract (f) (Key: 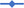: FBC values of compound A and compound B; 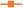: Additive line).

The combined effect of peppermint EO with other EOs and extracts was determined using the checkerboard method, and the FBC index was calculated using Equation 1. The FBC index results for *E. coli* are presented in Table 4 and Figure 1. Peppermint EO showed an additive effect against *E. coli* when combined with feijoa peel EO (FBC‐1.03), feijoa leaf EO (FBC‐1.06), and feijoa leaf extract (FBC‐0.62). The tested combinations showed an upward trend, with the concentrations tested lying above the additive line, as depicted in the isobologram (Figure 1c,d,f). However, when combined with thyme EO (FBC‐0.2813), lavender EO (FBC‐0.375), and feijoa peel extract (FBC‐0.3125), peppermint EO showed a synergistic effect with the isobologram showing a downward trend (Figure 1a,b,e). Notably, none of the tested combinations showed antagonistic action against *E. coli*. Similar results were observed for *L. monocytogenes*, where the combination of peppermint EO with thyme EO (FBC‐0.25), lavender EO (FBC‐0.5), and feijoa peel extract (FBC‐0.375) showed a synergistic effect (Figure 2a,b,e and Table 4), while feijoa peel EO, leaf EO, and feijoa leaf extracts showed an additive effect (Figure 2c,d,f and Table 4). However, the combination of peppermint EO and feijoa leaf EO showed indifferent/no interaction action against *L. monocytogenes* (Figure 2d and Table 4).

**TABLE 4:** FBC index and interactions of paired combinations of selected EOs and extracts against *Escherichia coli* and *Listeria monocytogenes*.

Bacteria	Combination	MBC (A alone)	MBC (B alone)	MBC (A in the presence of B)	MBC (B in the presence of A)	FBC A	FBC B	FBC	Interpretation
*E. coli*	PP + Lav	6.25	12.5	0.78125	3.125	0.125	0.25	0.375	Synergism
	PP + Thyme	6.25	12.5	0.1953125	3.125	0.0313	0.25	0.2813	Synergism
	PP + FjPl EO	6.25	50	0.1953125	50	0.0313	1	1.0313	Additive
	PP + FjLfEO	6.25	50	0.390625	50	0.0625	1	1.0625	Additive
	PP + FjPlEx	6.25	25	0.390625	6.25	0.125	0.25	0.3125	Synergism
	PP + FjLfEx	6.25	50	3.125	6.25	0.5	0.125	0.625	Additive
*L. monocytogenes*	PP + Lav	25	25	6.25	6.25	0.25	0.25	0.5	Synergism
	PP + Thyme	25	25	3.125	3.125	0.125	0.125	0.25	Synergism
	PP + FjPl EO	25	6.25	12.5	3.125	0.5	0.5	1	Additive
	PP + FjLfEO	25	3.125	3.125	3.125	0.125	1	1.125	Indifferent/no interaction
	PP + FjPlEx	25	12.5	3.125	3.125	0.125	0.25	0.375	Synergism
	PP + FjLfEx	25	25	12.5	12.5	0.5	0.5	1	Additive

Abbreviations: FjLfEO, Feijoa leaf essential oil; FjLfEx, Feijoa leaf extract.; FjPlEO, Feijoa peel essential oil; FjPlEx, Feijoa peel extract; Lav, Lavender; PP, Peppermint.

We found that the combination of peppermint/thyme demonstrated the highest potency with the lowest FBC index. One potential explanation for this observation is that thymol and carvacrol, which are major components of thyme and can disrupt the outer membrane of bacteria, may enhance the penetration of other EO molecules into the bacterial cell (Kim et al., 2021). The combination of peppermint/feijoa peel extract showed the second highest efficacy, followed by the combination of peppermint/lavender. It is worth noting that while peppermint, thyme, lavender, and feijoa peel extract individually displayed inhibitory effects at higher concentrations, the best synergistic effects were observed when these EOs and extracts were combined. The MBC value of each individual EO or extract was reduced significantly when used in combination with other EOs, indicating a combined antimicrobial effect. Peppermint EO exhibited a reduction in concentration by thirty times for *E. coli* and eight times for *L. monocytogenes*, while thyme EO showed a reduction of four times for *E. coli* and eight times for *L. monocytogenes*. In addition, peppermint EO combined with the lavender EO, and feijoa peel extract exhibited a reduction of four times in the inhibitory value for both *E. coli* and *L. monocytogenes*. These synergistic combinations were also tested against *B. cereus*, *St. aureus*, and *S. enterica* Typhimurium, and we found the combinations were effective at inhibiting all three pathogens. This result has further strengthened our confidence in our hypothesis that synergism effectively reduces the inhibitory concentration of the tested EOs and extracts.

This study supports previous research indicating that *Origanum compactum* and *Mentha piperita* have a synergistic effect against *E. coli*, with thymol, carvacrol, and menthol identified as the primary components responsible for this effect (El amrani et al., 2021). Similarly, Gallucci et al. (2009) found synergistic effects of menthol and thymol, in peppermint and thyme EO, respectively, against *B. cereus*, but an additive effect against *E. coli*. In another study (Bassole et al., 2010), the combination of menthol and carvacrol in peppermint and thyme EO, respectively, had an indifferent/no interactive effect against *L. monocytogenes*, but a synergistic effect against *E. coli*. Similarly, the combination of menthol and linalool in peppermint and lavender EO was additive against both bacteria. Previous studies have demonstrated additive or indifferent/no interactive effects of pure compounds of peppermint, thyme, and lavender EOs, but our study showed synergistic effects, indicating that minor components may play a vital antibacterial role, including interactions with each other. Peng et al. (2019a, 2019b) hypothesized that synergism could be attributed to factors such as aqueous solubility, lipophilic properties, and potency of functional groups, which all contribute to the antimicrobial activity of EO molecules. Other studies have also reported the enhanced inhibitory capacity of EOs such as peppermint, thyme, lavender, and their major components, including menthol, thymol, eugenol, and carvacrol (Bassole et al., 2010; Kim et al., 2021; Ouedrhiri et al., 2017; Soulaimani et al., 2021). However, this is the first study to investigate the combination of peppermint EO and feijoa peel extract and observe the synergistic effect of this combination against foodborne pathogens.

### 3.4 Time‐to‐kill kinetics

**FIGURE 3:**
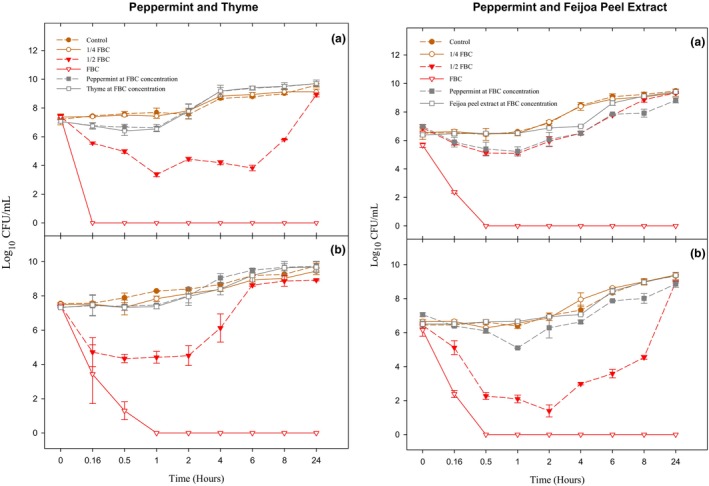
Time–kill curves of peppermint × thyme EO and peppermint EO × feijoa peel extract against *Escherichia coli* where (a) no BSA and (b) BSA.

Time‐to‐kill kinetics assays were conducted to validate the antimicrobial efficacy of the selected synergistic combinations of peppermint × thyme and peppermint × feijoa peel extract against the model organisms *E. coli* and *L. monocytogenes*. The combination of peppermint × thyme and peppermint × feijoa peel extract at FBC concentration resulted in significant declines (*p* < .001) in *E. coli* numbers and complete eradication at 10 min and 30 min, respectively (Figure 3). However, the same treatment required about 60 min to demonstrate a bactericidal effect (*p* < .001) when BSA was used to mimic the protein burden. When *E. coli* was treated with the peppermint × thyme combination at ½ FBC concentration, the population declined by 4 log_10_ at the 60‐min time point (*p* < .050), but we observed a gradual rise in the population throughout 24 h, indicating a bacteriostatic effect. This bacteriostatic effect (*p* > .050) was also observed when *E. coli* was treated with a combination of peppermint × feijoa peel extract at ½ FBC concentration. No significant differences (*p* > .050) were observed between all combinations at ¼ FBC concentration and single treatments compared to the control.

**FIGURE 4:**
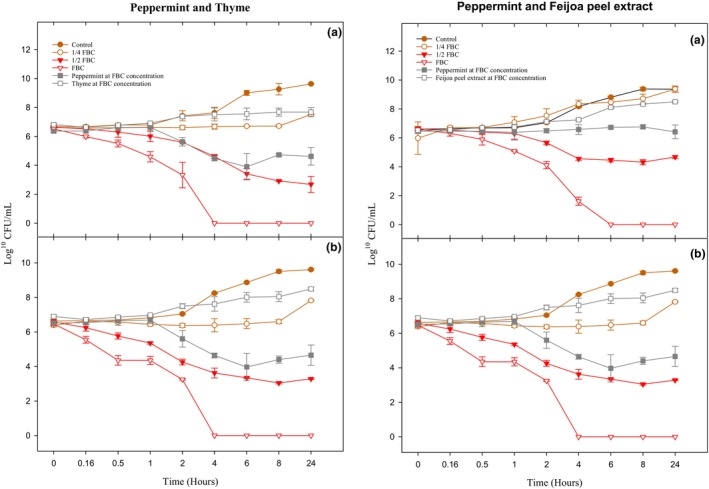
Time–kill curves of peppermint × thyme EO and peppermint EO × feijoa peel extract against *Listeria monocytogenes* where (a) no BSA and (b) BSA.

Interestingly, the application of peppermint alone at FBC concentration resulted in a significant (*p* < .050) reduction of *L. monocytogenes* compared to the control samples. However, the combinations at ¼ FBC concentration and the use of thyme and feijoa peel extract alone at FBC concentration did not demonstrate significant reductions (*p* > .050) in the bacterial numbers compared to the control. The population of *L. monocytogenes* gradually declined (*p* < .001) during the initial hours of treatment, and complete eradication was achieved at 4–6 h after treatment with a combination of peppermint × thyme and peppermint × feijoa peel extract at FBC concentration (Figure 4). However, at ½ FBC concentration, *L. monocytogenes* displayed a slightly different response compared to *E. coli*, with a slow population decline (3 log_10_ reduction) that remained constant over 24 h (*p* < .050). This finding is consistent with the study by Diarra et al. (2020), where a cranberry pomace extract at a lethal concentration eradicated *L. monocytogenes* after 6 h, whereas at a sublethal concentration, bacterial numbers reduced by 3 log_10_ and remained constant throughout 24 h. The findings indicated a concentration dependent effect, with higher concentrations leading to the rapid death of bacterial cell, highlighting a dose–response relationship. Other studies have also investigated the bactericidal effects of synergistic combinations of EOs on foodborne pathogens. For example, Bag and Chattopadhyay (2015) examined the effects of coriander and cumin EO, Barbosa et al. (2015) investigated the effectiveness of clove/cinnamon/rosemary/chamomile EOs, and Krasniewska et al. (2020) studied the impact of Spanish oreganum/Spanish marjoram/coriander EOs, where bactericidal effects were reported (Lim et al., 2023).

Furthermore, when treated only with peppermint, thyme EO, or feijoa peel extract, neither bacteriostatic nor bactericidal effects were observed for both *E. coli* and *L. monocytogenes*, underscoring the significance of the synergistic combinations.

## 4 CONCLUSIONS

In conclusion, our study demonstrated the potential synergistic effects of combinations of EOs and extracts against *E. coli* and *L. monocytogenes*, without any observed antagonistic effects. The combination of peppermint × thyme and peppermint × feijoa peel extract was the most effective, exhibiting a synergistic effect against both *E. coli* and *L. monocytogenes*, significantly reducing the concentration required to inhibit the pathogens when combined than the individual treatments of these pathogens. Moreover, the synergistic combination of peppermint × thyme and peppermint × feijoa peel extract also demonstrated effectiveness against *S. enterica* Typhimurium, *St. aureus*, and *B. cereus*, thus fulfilling the objective of developing a broad‐spectrum antimicrobial combination. The synergistic combinations eliminated *E. coli* within 10–30 min and reduced *L. monocytogenes* by ~3 log_10_ within 2 h, with complete eradication between 4 and 6 h, according to the time–kill curve.

Synergistic combinations allowed lower concentrations to be used, achieving the twin aims of reducing any potential undesirable organoleptic impact on food products while effectively controlling bacterial growth. These results highlight the potential of natural products as an alternative to traditional antimicrobial agents and pave the way for further research in this area. The use of plant EOs and extracts as a natural approach to enhance food safety and quality could have significant implications for public health and the food industry. Indeed, further research is warranted to comprehensively investigate the influence of volatile compounds present in EOs on the outcomes of synergistic assays. This entails delving into the intricate interactions between these volatile compounds and other components within the EOs, as well as their combined effects with extracts, a limitation in this study. Moreover, a deeper exploration into the underlying mechanisms of action of both individual treatments and paired combinations of EOs and extracts is necessary to fully understand these observations.

## AUTHOR CONTRIBUTIONS

**Manasweeta Angane:** Conceptualization (lead); data curation (lead); formal analysis (lead); investigation (lead); methodology (lead); validation (lead); writing – original draft (lead); writing – review and editing (lead). **Simon Swift:** Conceptualization (equal); methodology (equal); supervision (equal); writing – review and editing (equal). **Kang Huang:** Supervision (equal); writing – review and editing (equal). **Janesha Perera:** Methodology (supporting). **Xiao Chen:** Methodology (supporting). **Christine A. Butts:** Supervision (supporting); writing – review and editing (equal). **Siew Young Quek:** Conceptualization (lead); funding acquisition (lead); project administration (lead); supervision (lead); validation (equal); writing – review and editing (equal).

## FUNDING INFORMATION

This research is partially funded by The University of Auckland under the Press Account Number‐9448‐UOA‐MANG207, and the Food and Health Programme Seed Grant (4200‐UOA‐48422‐A8AN).

## CONFLICT OF INTEREST STATEMENT

None declared.

## Supporting information


Appendix S1

## Data Availability

The data that support the findings of this study are available from the corresponding author upon reasonable request.
