# Supplementary material for: Synergistic antimicrobial interaction of plant essential oils and extracts against foodborne pathogens
Source: Food Sci Nutr. 2023 Nov 22;12(2):1189–206. doi: 10.1002/fsn3.3834 (PMC10867478; doi:10.1002/fsn3.3834)
Supplement: Supplementary file 1 — Appendix S1 [file FSN3-12-1189-s001.pptx]

## Slide 1
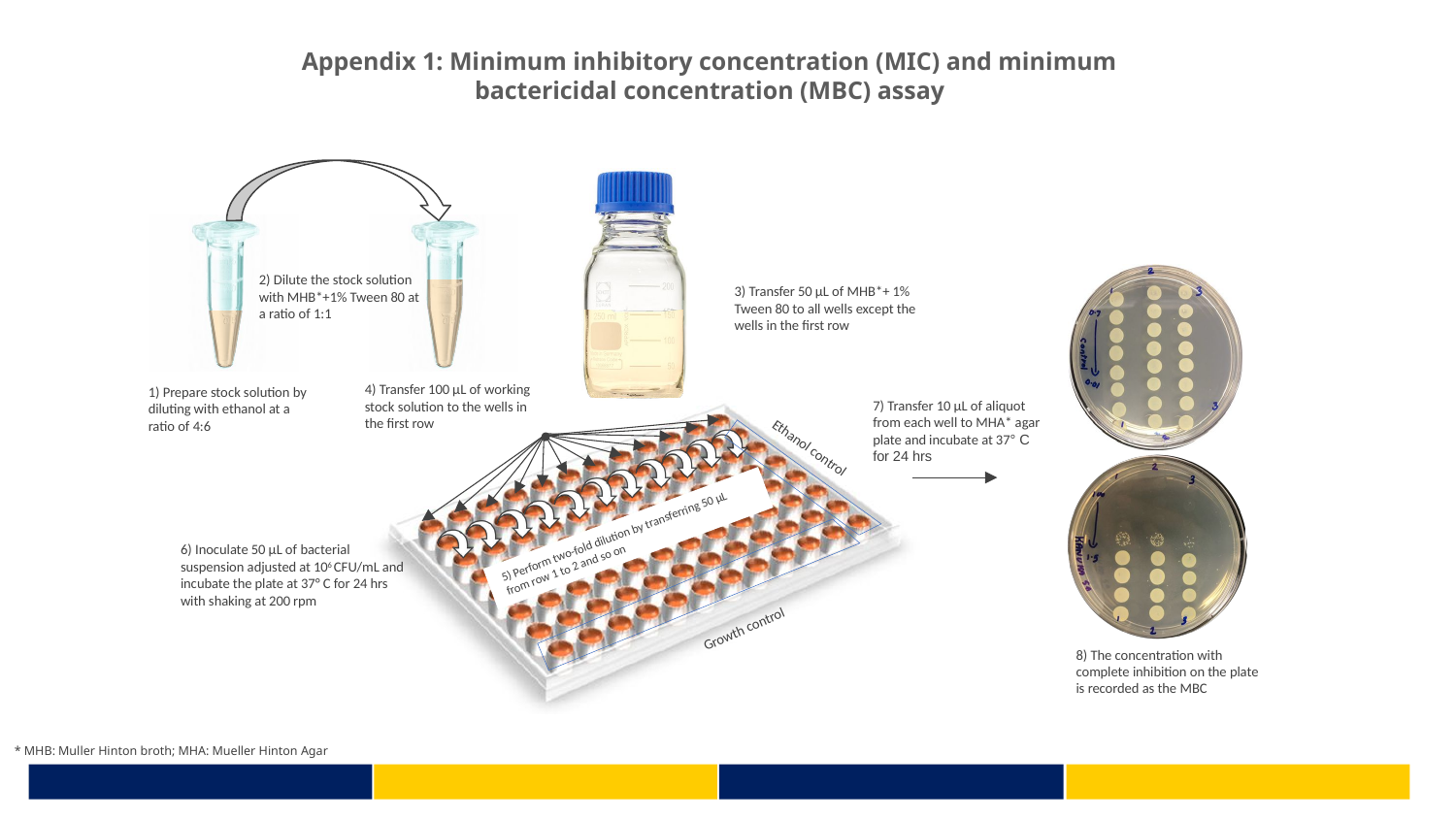

Appendix 1: Minimum inhibitory concentration (MIC) and minimum bactericidal concentration (MBC) assay
1) Prepare stock solution by diluting with ethanol at a ratio of 4:6
2) Dilute the stock solution with MHB*+1% Tween 80 at a ratio of 1:1
3) Transfer 50 µL of MHB*+ 1% Tween 80 to all wells except the wells in the first row
4) Transfer 100 µL of working stock solution to the wells in the first row
7) Transfer 10 µL of aliquot from each well to MHA* agar plate and incubate at 37° C for 24 hrs
Ethanol control
5) Perform two-fold dilution by transferring 50 µL from row 1 to 2 and so on
6) Inoculate 50 μL of bacterial suspension adjusted at 106 CFU/mL and incubate the plate at 37° C for 24 hrs with shaking at 200 rpm
Growth control
8) The concentration with complete inhibition on the plate is recorded as the MBC
* MHB: Muller Hinton broth; MHA: Mueller Hinton Agar

## Slide 2
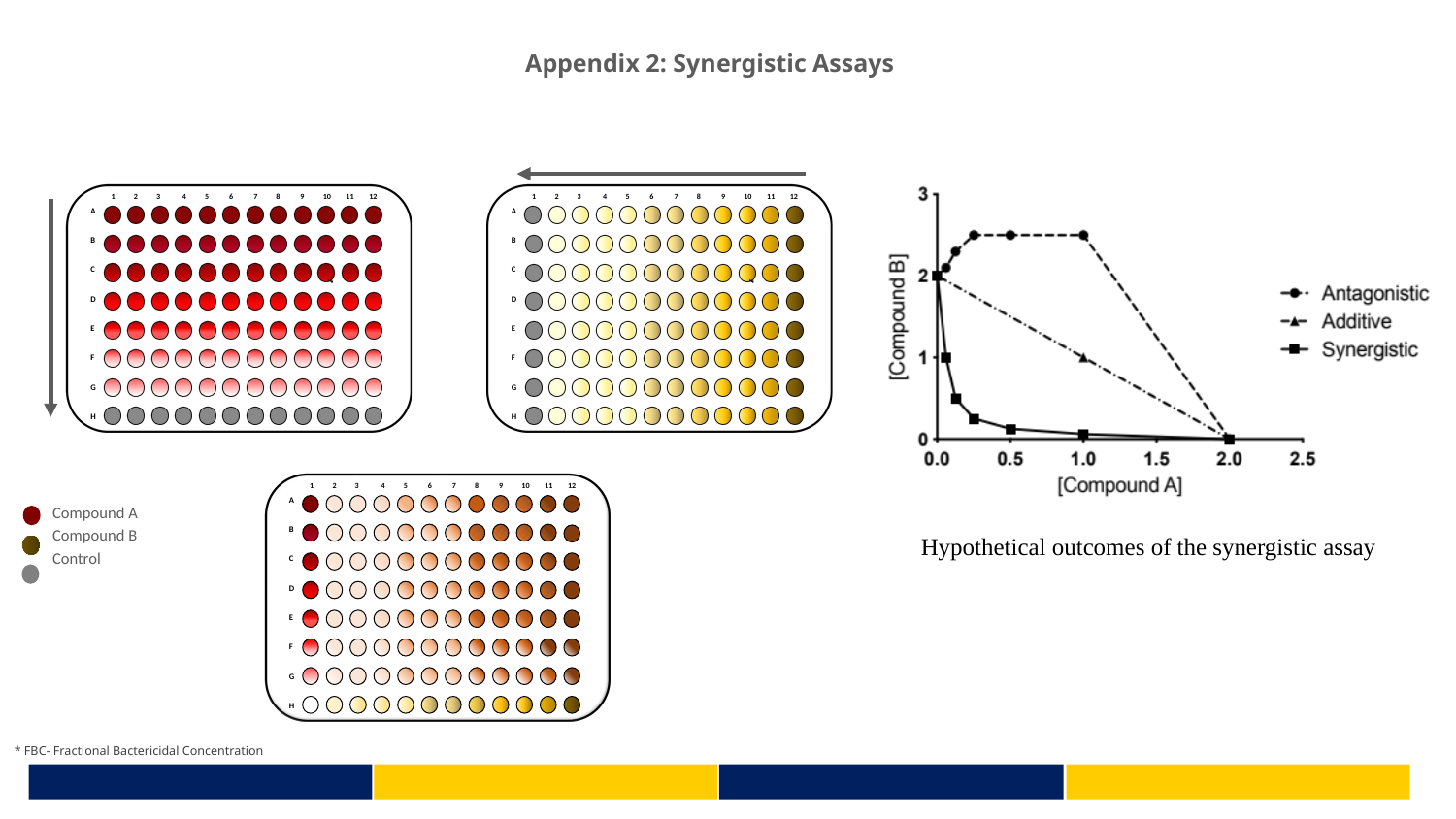

Appendix 2: Synergistic Assays
 Compound A
 Compound B
 Control
Hypothetical outcomes of the synergistic assay
* FBC- Fractional Bactericidal Concentration

## Slide 3
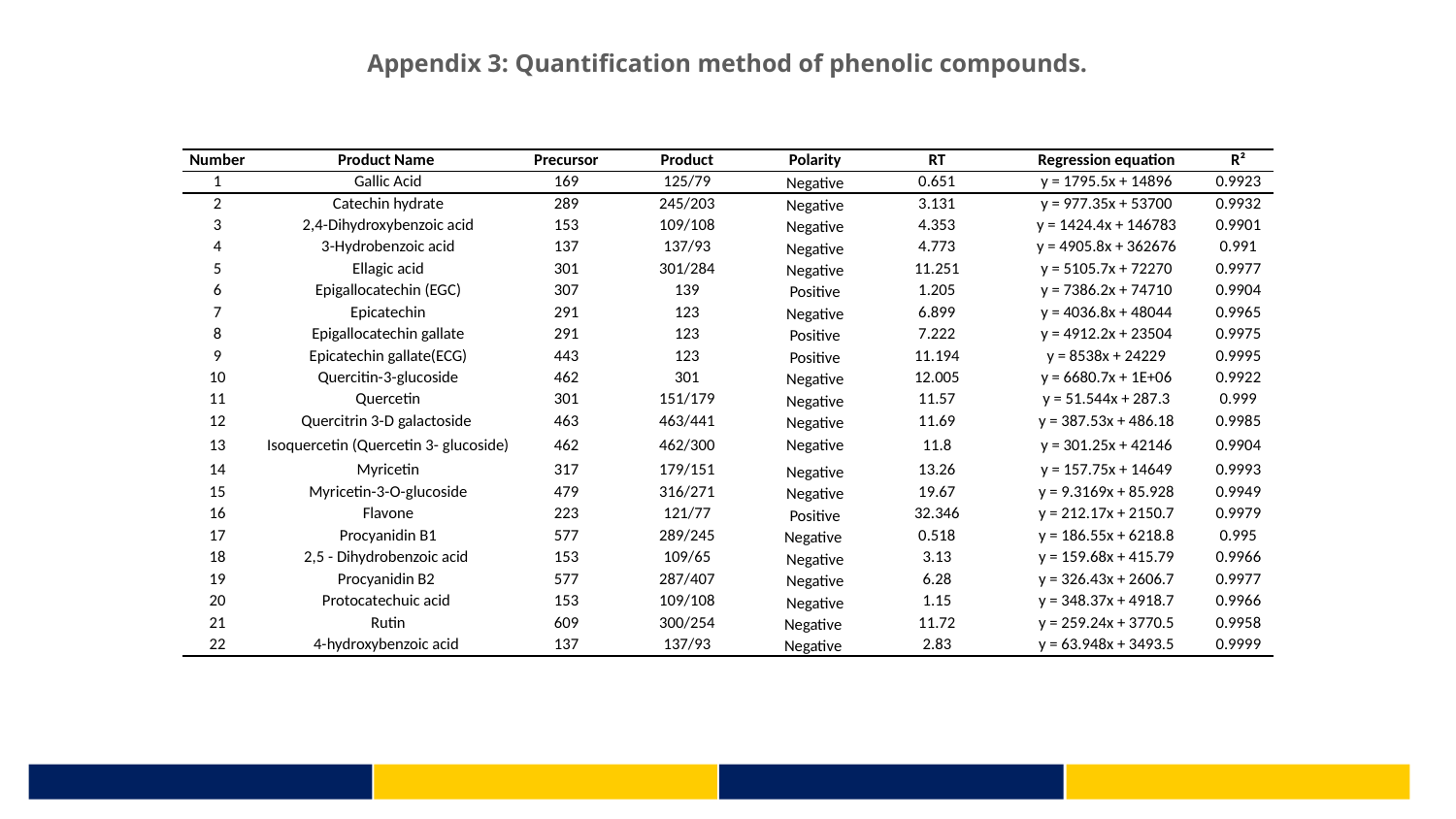

Appendix 3: Quantification method of phenolic compounds.
| Number | Product Name | Precursor | Product | Polarity | RT | Regression equation | R² |
| --- | --- | --- | --- | --- | --- | --- | --- |
| 1 | Gallic Acid | 169 | 125/79 | Negative | 0.651 | y = 1795.5x + 14896 | 0.9923 |
| 2 | Catechin hydrate | 289 | 245/203 | Negative | 3.131 | y = 977.35x + 53700 | 0.9932 |
| 3 | 2,4-Dihydroxybenzoic acid | 153 | 109/108 | Negative | 4.353 | y = 1424.4x + 146783 | 0.9901 |
| 4 | 3-Hydrobenzoic acid | 137 | 137/93 | Negative | 4.773 | y = 4905.8x + 362676 | 0.991 |
| 5 | Ellagic acid | 301 | 301/284 | Negative | 11.251 | y = 5105.7x + 72270 | 0.9977 |
| 6 | Epigallocatechin (EGC) | 307 | 139 | Positive | 1.205 | y = 7386.2x + 74710 | 0.9904 |
| 7 | Epicatechin | 291 | 123 | Negative | 6.899 | y = 4036.8x + 48044 | 0.9965 |
| 8 | Epigallocatechin gallate | 291 | 123 | Positive | 7.222 | y = 4912.2x + 23504 | 0.9975 |
| 9 | Epicatechin gallate(ECG) | 443 | 123 | Positive | 11.194 | y = 8538x + 24229 | 0.9995 |
| 10 | Quercitin-3-glucoside | 462 | 301 | Negative | 12.005 | y = 6680.7x + 1E+06 | 0.9922 |
| 11 | Quercetin | 301 | 151/179 | Negative | 11.57 | y = 51.544x + 287.3 | 0.999 |
| 12 | Quercitrin 3-D galactoside | 463 | 463/441 | Negative | 11.69 | y = 387.53x + 486.18 | 0.9985 |
| 13 | Isoquercetin (Quercetin 3- glucoside) | 462 | 462/300 | Negative | 11.8 | y = 301.25x + 42146 | 0.9904 |
| 14 | Myricetin | 317 | 179/151 | Negative | 13.26 | y = 157.75x + 14649 | 0.9993 |
| 15 | Myricetin-3-O-glucoside | 479 | 316/271 | Negative | 19.67 | y = 9.3169x + 85.928 | 0.9949 |
| 16 | Flavone | 223 | 121/77 | Positive | 32.346 | y = 212.17x + 2150.7 | 0.9979 |
| 17 | Procyanidin B1 | 577 | 289/245 | Negative | 0.518 | y = 186.55x + 6218.8 | 0.995 |
| 18 | 2,5 - Dihydrobenzoic acid | 153 | 109/65 | Negative | 3.13 | y = 159.68x + 415.79 | 0.9966 |
| 19 | Procyanidin B2 | 577 | 287/407 | Negative | 6.28 | y = 326.43x + 2606.7 | 0.9977 |
| 20 | Protocatechuic acid | 153 | 109/108 | Negative | 1.15 | y = 348.37x + 4918.7 | 0.9966 |
| 21 | Rutin | 609 | 300/254 | Negative | 11.72 | y = 259.24x + 3770.5 | 0.9958 |
| 22 | 4-hydroxybenzoic acid | 137 | 137/93 | Negative | 2.83 | y = 63.948x + 3493.5 | 0.9999 |
